# Quantitative, noninvasive assessment of intra‐ and extraocular perfusion by contrast‐enhanced ultrasonography and its clinical applicability in healthy dogs

**DOI:** 10.1111/vop.12648

**Published:** 2019-02-17

**Authors:** Klaas‐Ole Blohm, Katharina M. Hittmair, Alexander Tichy, Barbara Nell

**Affiliations:** ^1^ Department for Companion Animals and Horses, Ophthalmology Service University of Veterinary Medicine Vienna Austria; ^2^ Department for Companion Animals and Horses, Diagnostic Imaging University of Veterinary Medicine Vienna Austria; ^3^ Department of Biomedical Sciences University of Veterinary Medicine Vienna Austria

**Keywords:** canine eyes, conscious dogs, contrast‐enhanced ultrasonography, perfusion parameters, SonoVue®, sulfur hexafluoride

## Abstract

**Objective:**

To assess quantitative perfusion of intra‐ and extraocular regions of interest (ROIs) in conscious, healthy dogs utilizing contrast‐enhanced ultrasonography (CEUS); to compare varying enhancement with the first and second bolus injection and in the right and left eye; and to determine the most appropriate examination time.

**Procedures:**

Gray scale ultrasonography and contrast harmonic imaging using sulfur hexafluoride were performed randomly assigned in both eyes in 10 university‐owned beagles. Perfusion parameters including slope time, time to peak (TTP), peak intensity (PI), and area under the curve (AUC) were measured at individually drawn ROIs (retrobulbar cone = ROI 1, choroid‐retina complex = ROI 2, medial = ROI 3, and lateral anterior uvea = ROI 4).

**Results:**

Time‐intensity curve parameters revealed no significant differences in eyes examined by the first or second bolus injection (*P* > 0.05) or in the right or left eye (*P* > 0.05). Pooled data from all eyes were analyzed. Peak intensity of ROI 2 was significantly higher compared to all other ROIs (*P* < 0.001). Area under the curve at ROI 2 was significantly higher compared to all other ROIs (*P* < 0.05), and AUC at ROI 1 was significantly higher than at ROI 4 (*P* < 0.05). No significant differences in TTP were observed between different ROIs (*P* > 0.05). Ratios relative to different ROI sizes showed fastest enhancement in the retrobulbar cone and most intense perfusion in the anterior uveal regions. The first minute after contrast injection provided the highest diagnostic value.

**Conclusion:**

Quantitative perfusion in nondiseased canine eyes revealed consistent parameters. Application of standardized CEUS protocols may be a promising diagnostic tool to differentiate ocular lesions.

## 1 INTRODUCTION

Diagnostic properties of ophthalmic examination techniques are limited due to the eye's anatomy and can be afflicted by degenerative or post‐traumatic^1^, ^2^, ^3^, ^4^ opacifications of ocular media. Brightness mode (B‐mode) ultrasonography remains the widely available,^4^, ^5^, ^6^ less expensive,^4^, ^6^ and noninvasive^5^, ^6^ diagnostic imaging method of choice^4^, ^7^ with contrast‐enhanced ultrasonography (CEUS) providing additional advantages.^8^, ^9^, ^10^

Miszalok et al^11^ realized promising potential in ultrasonographic assessment of blood flow dynamics in canine eyes by means of contrast enhancement. More recently, differentiation of retinal detachment from vitreous membrane was facilitated by CEUS in humans,^12^, ^13^ cats, and dogs.^9^ In human ophthalmology, very good inter‐observer agreement and diagnostic accuracy of CEUS for retinal/choroidal detachment and/or intraocular masses^1^ and increased visualization of retrobulbar and ciliary arteries after intravenous contrast medium infusion were identified.^14^ Physiologic and experimentally impaired choroidal perfusion was distinguished in rabbits^15^ utilizing contrast‐enhanced harmonic ultrasonography, while fundamental high‐frequency contrast‐enhanced ultrasound correlated with histologic uveal melanoma size and vascularity in mice^16^ and rabbits.^17^ The use of CEUS is increasing in oncology patients and the recent focus of research lies in the diagnostic significance of quantitative time‐intensity curve (TIC) parameters.

Hong et al^8^ described perfusion using perfluorobutane contrast medium (Sonazoid®) in the right eyes of healthy, anesthetized beagles. Although sulfur hexafluoride microbubbles (SonoVue®) proved to be feasible evaluating various ocular pathologies,^1^, ^9^, ^10^, ^13^, ^18^, ^19^, ^20^ reports assessing physiologic perfusion of intra‐ and extraocular tissues in dogs are lacking.

The primary aim of this study was to assess quantitative perfusion parameters of the retrobulbar cone, the choroid and retina, and the anterior uvea in healthy dogs without chemical restraint. We hypothesized that the sequence of CEUS examinations has no significant impact on contrast enhancement and that the opposite eye can serve as an intraindividual in vivo reference. An additional objective was to determine the most appropriate application time of ocular CEUS.

## 2 MATERIALS AND METHODS

### 2.1 Animals studied

This prospective, randomized clinical study included ten university‐owned, male (seven neutered, three intact), beagles (n = 20 eyes) with a monitored health status. Prior to and during this trial, none of the dogs were used for other examinations that might have altered the evaluated ocular parameters. Approval for this study was obtained by the institution's Ethics and Animal Welfare Committee and the national authority (GZ: BMWFW‐68.205/0039‐WF/V/3b/2017).

A complete physical examination including body condition scoring^21^ was performed and hematocrit and total protein values were determined. Ophthalmic examination included Schirmer test 1 (Teststreifen, MSD, Unterschleissheim, Germany), menace response, pupillary light reflexes, dazzle reflex, slit lamp biomicroscopy (Kowa SL‐15®; Kowa, Tokyo, Japan), fluorescein staining (Fluorotouch Ophthalmic Strips®; Eickemeyer, Tuttlingen, Germany), tonometry (TonoVet®; icare, Vantaa, Finland), and indirect funduscopy (Keeler Vantage®; Keeler Instruments Inc, Broomall, PA) after pupillary dilation with tropicamide (Mydriatikum®; Agepha, Senec, Slovakia). Following topical 0.4% oxybuprocaine hydrochloride instillation (Novain®; Agepha, Vienna, Austria), ocular B‐mode ultrasonography**,** using a 12‐5 MHz linear transducer (iU22 Philips®; Philips, Bothell, WA), was performed to examine the globe and to rule out orbital pathologies. Exclusion criteria were systemic and ophthalmic abnormalities based on these examinations. The dogs underwent physical and ophthalmic re‐evaluation 24 hours after CEUS. All procedures were conducted by the first author (KOB) under supervision of a board‐certified ophthalmologist (BN).

### 2.2 Contrast‐enhanced ultrasonography

**Figure 1:**
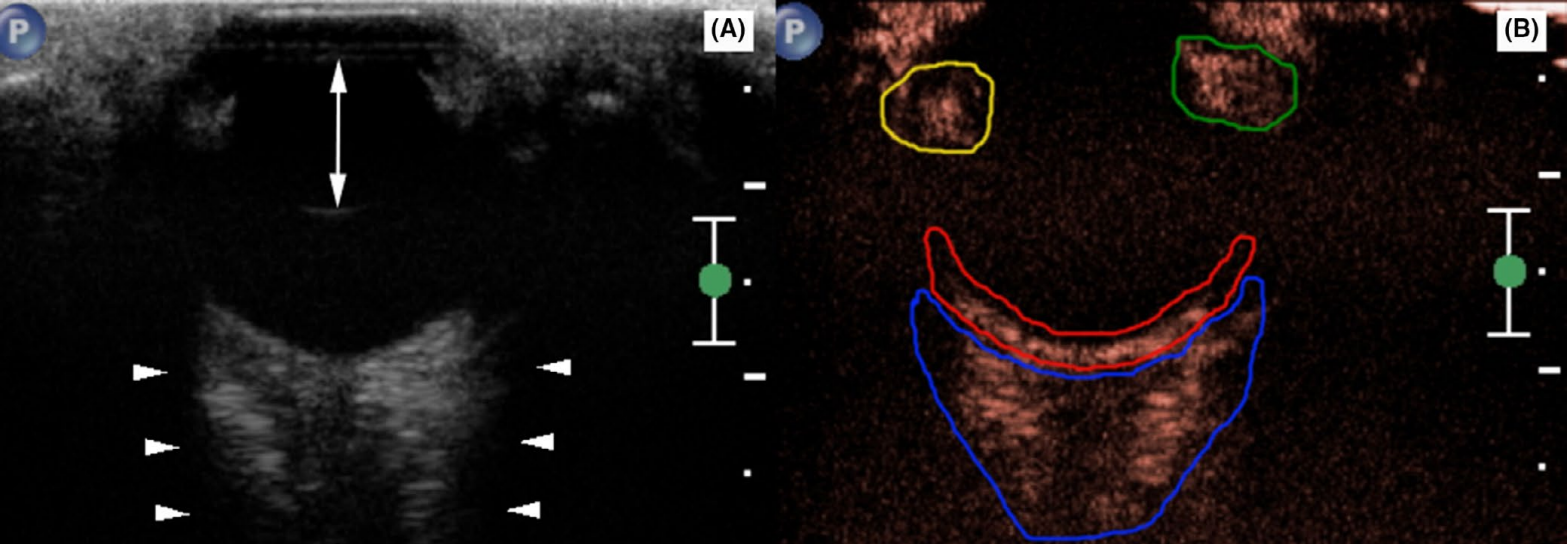
Representative ocular CEUS split screen. (A) The gray scale image displays anatomic landmarks: lens capsule borders (two‐sided arrow) and bony orbit's acoustic shadowing (arrowheads). (B) Note specified regions of interest (ROIs) in the contrast image from posterior to anterior: retrobulbar cone (blue), choroid‐retina complex (red), medial (yellow) and lateral (green) anterior uvea. Marker position ‘P’ (upper left corner) was medial. [Correction added on March 1, 2019 after online publication: Figure 1 has been updated.]

The right and left eye were examined using sulfur hexafluoride contrast medium (SonoVue®, Lot‐number: 16A029C; Bracco, Milan, Italy) bolus injection with the order chosen randomly by coin toss. Evaluation of the opposite eye was performed at least 5 minutes later. After topical application of 0.4% oxybuprocaine hydrochloride, conscious animals were positioned in lateral recumbency with the head supported in a steady position by manual restraint. Positioning was switched to the opposite lateral recumbency during the 5 minute interval. Contrast harmonic imaging was performed utilizing the same 12‐5 MHz linear array transducer (iU22 Philips®). A transcorneal, horizontal plane of the globe visualized echoes from the anterior and posterior lens capsule and the surface of the bony orbit as anatomic references (Figure 1A). This probe orientation was attempted for each entire sequence. Bolus injections of SonoVue® (0.03 mL/kg bodyweight^20^, ^22^, ^23^, ^24^, ^25^) were administered within 1 to 2 seconds^25^ via an indwelling right cephalic vein catheter (Vasofix 22 G®; B. Braun Melsungen AG, Melsungen, Germany) followed by 5 mL NaCl 0.9% flush (B. Braun Melsungen AG, Melsungen, Germany). Both syringes were attached to a three‐way stopcock, and contrast medium was injected through the port in the direction of the catheter extension (B. Braun 30 cm luer‐lock, B. Braun Melsungen AG, Melsungen, Germany) with the right front leg extended. The recording timer was set to 120 seconds (1178 frames) for each video, which started simultaneously with SonoVue® injection. The adjustable ultrasound settings including mechanical index (MI = 0.1), depth (3 cm), focus (1.5 cm), and pulse repetition frequency at 10 Hz with a 85% gain value were fixed for each examination. All imaging procedures were carried out by the same examiner (KOB) supervised by a radiologist with comprehensive expertise in ocular and contrast ultrasonography (KH).

### 2.3 Regions of interest

All CEUS examinations were analyzed using dedicated quantification software (QLAB Release 10.7®; Philips Austria GmbH, Vienna, Austria). Specific regions of interest (ROIs) delineated four anatomic structures avoiding inclusion of adjacent tissues (Figure 1B). Time‐intensity curve quality and quantitative blood flow measurements were obtained for the retrobulbar cone (ROI 1), the choroid‐retina complex (ROI 2), the medial (ROI 3), and lateral (ROI 4) anterior uvea.

### 2.4 Quantitative assessment

**Figure 2:**
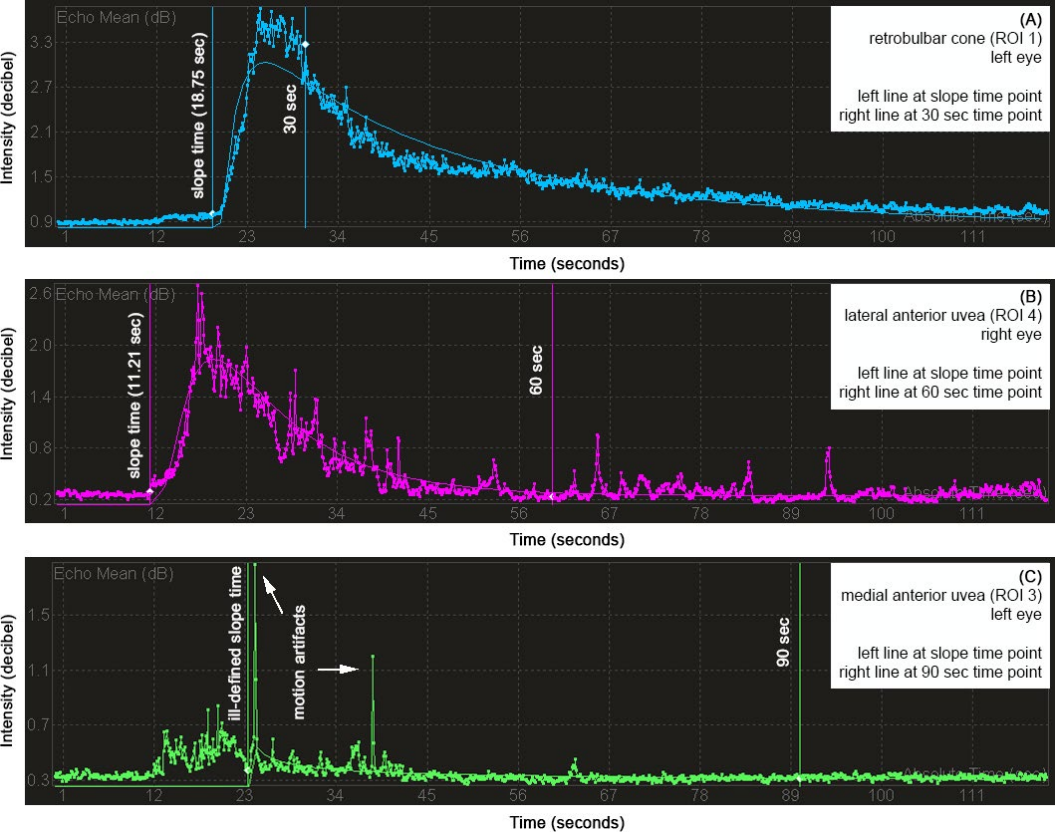
Time‐intensity curve quality was scored (A) excellent, if minimal oscillation of signals and no motion artifacts were present, (B) good, if variations of signals and amount of motion artifacts were mild to moderate, (C) insufficient, if acquired data did not enable application of curve fit or if marked motion artifacts were present. (A‐B) Local density random walk (LDRW) model resulted in a smooth graph for quantitative assessment of perfusion parameters

The TICs include baseline tissue echoes and postcontrast intensities (contrast enhancement). Signal detection was semiquantitatively scored. A rapid, marked amplitude (wash‐in), and a continuous decline (wash‐out) characterized appropriate TIC quality (Figure 2). Oscillations of signal intensities on TICs were minimal with no motion artifacts and rated excellent or mild to moderate with few and low‐intensity motion artifacts and rated good. Time‐intensity curve quality not suitable for the local density random walk (LDRW) curve fit or TICs with numerous or high‐intensity motion artifacts were graded insufficient. Cine‐loops with excellent or good contrast‐dilution acquisition contributed to statistical analysis, whereas insufficient TICs in one or more ROIs led to exclusion of this dog's imaging set.

The region size in square millimeters (mm^2^) was determined for each outlined ROI, and LDRW curve fit model was accomplished to generate time to peak (TTP) in seconds (sec), peak intensity (PI) in decibel (db), and area under the curve (AUC) as db x sec. Time to PI (maximum microbubble concentration) is determined by TTP, whereas AUC represents the amount of microbubbles passing through a tissue section of interest over time. The TTP: region size ratios calculated ROI‐specific time for maximal signal intensity and the AUC: region size ratios quantified ROI‐specific perfusion per mm^2^ over time.

For advanced assessment, individually performed intensity measurements, at 0 sec, at slope time, at 30, 60, 90, and 120 sec time points, were applied for each ROI. The slope time in sec was defined as the time point identifying presence and increase of microbubble concentration (Figure 2). One author (KOB) performed all measurements which were reviewed by a board‐certified ophthalmologist (BN) and an ultrasound specialist (KH).

### 2.5 Qualitative assessment

The real‐time enhancement pattern of all ROIs was qualitatively evaluated. Criteria were homogeneity and distribution of contrast enhancement and the relative postcontrast intensity of the different ROIs. Qualitative assessment was performed by one examiner (KOB).

### 2.6 Statistical analysis

Normality for metric parameters was evaluated with a Kolmogorov‐Smirnov test. Data of descriptive statistics were expressed as mean ± standard deviation (SD). Correlations were investigated using Pearson's correlation coefficient. Measurements generated separately for both eyes by random order were analyzed independently. The region size, slope time, TTP, PI, and AUC were compared for all ROIs with regard to first or second bolus injection and right or left eye performing mixed model analysis.

Differences between measurements of specified ROIs were investigated by one‐way ANOVA using Bonferroni's alpha‐correction and Scheffé’s alpha‐correction as post hoc procedures. As quantitative perfusion parameters are related to the varying region sizes, ratios were calculated (slope time: region size, TTP: region size, PI: region size, AUC: region size). Ratios of the different ROIs were compared using a mixed model approach with Bonferroni's alpha‐correction procedure.

Time point investigations for all ROIs were performed by linear model with Bonferroni's alpha‐correction procedure. A value of *P* < 0.05 was considered significant for all statistical analyses. Calculations were performed using IBM SPSS v24® (IBM Corp., Armonck, NY).

## 3 RESULTS

### 3.1 Animals studied and time‐intensity curve quality

Mean age ± SD of the study population was 27.6 ± 8.7 months (range 19‐37 months). Weights and body condition scores ranged from 12.4 to 18.0 kg (mean 15.2 ± SD 2.3 kg) and from 4/9 to 6/9 (mean 5/9), respectively. Physical and ophthalmic examinations, hematocrit (mean 48.8% ± SD 2.4%), total protein (mean: 6.8 g/dL ± SD 0.2 g/dL), and ocular B‐mode ultrasonography were within normal limits for all animals (n = 10 dogs, n = 20 eyes). Age and bodyweight showed a significant positive correlation (*r* = 0.906; *P* < 0.001).

Time‐intensity curve quality was graded excellent in 46/80 (57.5%) ROIs while 34/80 (42.5%) ROIs were evaluated as good. Initial CEUS sequences of 2/10 beagles (20%) were excluded due to insufficient scoring in 2/8 ROIs and 5/8 ROIs, respectively. These two beagles had an additional CEUS examination which revealed appropriate TIC quality. The corrected distribution of all ROIs yielded 57.3% (55/96), 35.4% (34/96), 7.3% (7/96) with excellent, good, and insufficient grading, respectively. Insufficient contrast detection in one ROI implicated exclusion of this imaging sequence and, therefore, the entire CEUS examination (eight ROIs). Consequently, 80 ROIs were included. No systemic or ocular adverse effects were observed. Physical and ophthalmic re‐examinations 24 hours after CEUS revealed no abnormalities.

### 3.2 First and second bolus injection

**Figure 3:**
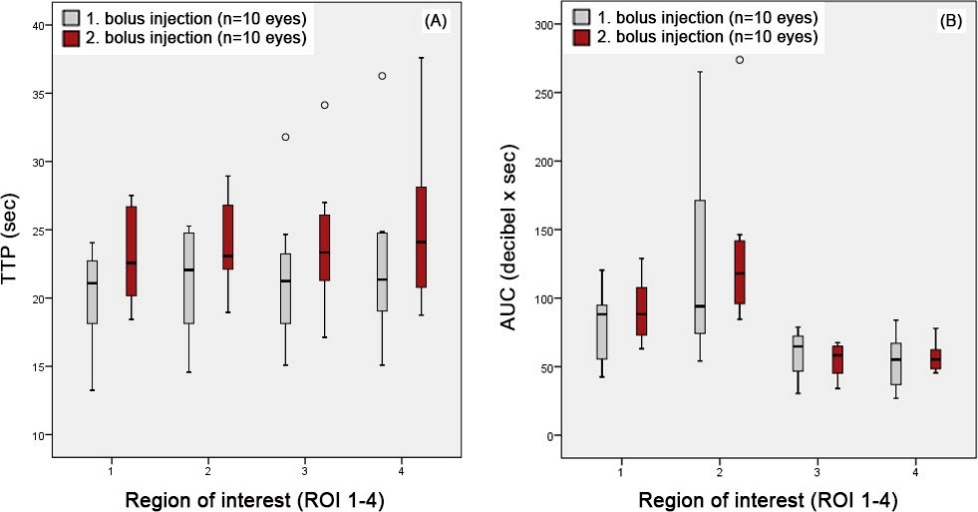
(A) Time to peak (TTP) after first and second bolus injections. The overall time frame was similar even though TTP of second bolus injections was consistently increased. (B) Area under the curve (AUC) at first and second bolus injection. Second bolus injections revealed less variation of AUC, particularly at ROI 2. (A‐B) Differences detected by injection order were not statistically significant (*P* > 0.05) for any ROI (x‐axis: 1 = retrobulbar cone, 2 = choroid‐retina complex, 3 = medial‐, 4 = lateral anterior uvea)

All eyes that underwent CEUS with the second bolus injection (n = 10 eyes) trended towards an increased slope time, PI, and AUC. Following second bolus injection, TTP showed consistently increased minimum, maximum, and mean values in all ROIs (Figure 3A). Area under curve at ROI 3 (first bolus injection: 59.58 ± 17.15 db x sec, second bolus injection: 55.32 ± 11.30 db x sec) was the only parameter that did not increase (Figure 3B). Differences for all perfusion parameters reached no statistical significance when comparing eyes examined with first or second bolus injection (*P* > 0.05).

### 3.3 Right and left eye

All right eyes revealed a consistently shorter TTP regarding all ROIs compared with the left eyes (Table 1). No statistically significant difference in perfusion parameters was observed between the right and left eyes (*P* > 0.05).

**Table 1:** Time to peak (TTP) in seconds (sec) laterality comparison

	TTP (sec) right eyes (n = 40)			TTP (sec) left eyes (n = 40)		
	Min.	Max.	Mean ± SD	Min.	Max.	Mean ± SD
Retrobulbar cone (ROI 1)	13.25	27.41	19.90 ± 4.24	18.44	27.51	23.07 ± 2.85
Choroid‐retina complex (ROI 2)	14.57	28.43	20.71 ± 4.08	18.95	28.94	24.18 ± 2.77
Medial anterior uvea (ROI 3)	15.08	26.08	20.79 ± 3.57	17.12	34.13	24.38 ± 5.41
Lateral anterior uvea (ROI 4)	15.08	28.12	21.60 ± 3.69	18.75	37.60	26.68 ± 6.89

n, number of ROIs; ROI, region of interest.

### 3.4 Quantitative assessment

As there were no significant differences concerning order of bolus injections or significant impact of laterality, pooled data served for further analysis (Table 2). Region size of ROI 1, which included the extraocular muscles, orbital fat, and optic nerve, was significantly larger than that of all other ROIs (*P* < 0.001), and the size of ROI 2 was significantly larger compared to ROI 3 and ROI 4 (*P* < 0.001).

**Table 2:** Descriptive statistics for quantitative perfusion parameters in all ROIs for n = 20 eyes in n = 10 clinically normal, unsedated beagles

	Retrobulbar cone (ROI 1)			Choroid‐retina complex (ROI 2)			Med. anterior uvea (ROI 3)			Lat. anterior uvea (ROI 4)		
	Min.	Max.	Mean ± SD	Min.	Max.	Mean ± SD	Min.	Max.	Mean ± SD	Min.	Max.	Mean ± SD
Region size (mm^2^)	160.14	175.26	168.42 ± 4.02^b,c,d^	43.07	60.68	46.65 ± 4.18^a,c,d^	18.56	22.00	20.44 ± 0.94^a,b^	18.26	21.49	19.92 ± 0.73^a,b^
Slope time (sec)	7.85	20.58	13.44 ± 3.25	7.44	20.58	13.00 ± 3.42	8.46	29.04	15.42 ± 4.94	8.35	21.50	14.50 ± 3.50
TTP (sec)	13.25	27.51	21.48 ± 3.87	14.57	28.94	22.44 ± 3.84	15.08	34.13	22.58 ± 4.82	15.08	37.60	24.14 ± 6.00
PI (db)	1.07	4.35	2.42 ± 0.85^b^	1.63	6.58	3.92 ± 1.33^a,c,d^	0.83	3.72	1.90 ± 0.74^b^	1.08	4.06	2.11 ± 0.74^b^
AUC (db x sec)	42.47	128.94	85.27 ± 22.73^b,d^	54.12	273.87	126.07 ± 58.75^a,c,d^	30.41	78.84	57.45 ± 14.31^b^	26.96	83.95	55.74 ± 14.67^a,b^
Slope time: region size ratio (sec/mm^2^)	0.05	0.12	0.08 ± 0.02^b,c,d^	0.15	0.45	0.28 ± 0.08^a,c,d^	0.43	1.42	0.75 ± 0.24^a,b^	0.43	1.03	0.73 ± 0.17^a,b^
TTP: region size ratio (sec/mm^2^)	0.08	0.17	0.13 ± 0.23^b,c,d^	0.25	0.63	0.49 ± 0.10^a,c,d^	0.70	1.67	1.11 ± 0.24^a,b^	0.74	1.89	1.22 ± 0.32^a,b^
PI: region size ratio (db/mm^2^)	0.01	0.03	0.01 ± 0.00^b,c,d^	0.03	0.14	0.08 ± 0.03^a^	0.04	0.19	0.09 ± 0.04^a^	0.05	0.21	0.11 ± 0.04^a^
AUC: region size ratio (db x sec/mm^2^)	0.27	0.74	0.50 ± 0.13^b,c,d^	1.18	6.03	2.71 ± 1.22^a^	1.54	3.87	2.82 ± 0.71^a^	1.35	4.33	2.81 ± 0.78^a^

Min., lowest measurement; Max., highest measurement; n, number of eyes and animals, respectively; ROI, region of interest; SD, standard deviation.

Superscript letters a, b, c, d indicate significant difference (a) to ROI 1, (b) to ROI 2, (c) to ROI 3, (d) to ROI 4 at *P* < 0.05.

**Figure 4:**
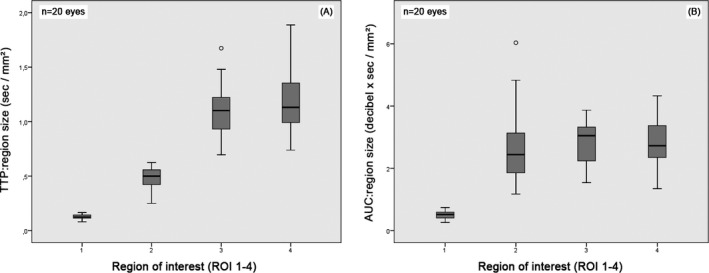
Calculated TTP: region size and AUC: region size ratios for standardized perfusion comparison of ROIs (x‐axis: 1 = retrobulbar cone, 2 = choroid‐retina complex, 3 = medial‐, 4 = lateral anterior uvea) revealed (A) fastest contrast enhancement from posterior to anterior structures and (B) highest perfusion intensity in the anterior uveal regions

There were no statistically significant differences between all ROIs regarding slope time and TTP (*P* > 0.05). Peak intensity and AUC at ROI 2 were significantly higher compared to ROI 1 (PI: *P* < 0.001; AUC: *P* = 0.001) and to ROI 3 and ROI 4 (PI: *P* < 0.001; AUC: *P* < 0.001). AUC at ROI 1 was significantly higher than at ROI 4 (*P* < 0.05). Perfusion parameters are related to the region size. Consequently**,** ratios per mm^2^ were calculated to determine representative ROI‐specific parameters in a standardized manner (Table 2). Slope time: region size (*P* < 0.001) and TTP: region size ratios (*P* < 0.001) of ROI 1 and ROI 2 were significantly lower compared to ROI 3 and ROI 4, while both anterior uveal regions did not vary significantly to each other (*P* > 0.05). Peak intensity: region size (*P* < 0.001) and AUC: region size ratios (*P* < 0.001) of ROI 1 were significantly lower than those of ROI 2 and both anterior uveal regions (PI: *P* < 0.001; AUC: *P* < 0.001). The calculated ratios for ROI 1 (TTP: 0.13 ± 0.23 sec/mm^2^; AUC: 0.50 ± 0.13 db x sec/mm^2^), ROI 2 (TTP: 0.49 ± 0.10 sec/mm^2^; AUC: 2.71 ± 1.22 db x sec/mm^2^), ROI 3 (TTP: 1.11 ± 0.24 sec/mm^2^; AUC: 2.82 ± 0.71 db x sec/mm^2^), and ROI 4 (TTP: 1.22 ± 0.32 sec/mm^2^; AUC: 2.81 ± 0.78 db x sec/mm^2^) demonstrated significantly less time for contrast enhancement per mm^2^ and significantly less intense perfusion per mm^2^ from the posterior to anterior ocular tissues (Figure 4).

### 3.5 Qualitative assessment

The qualitative evaluation of the retrobulbar cone revealed a heterogeneous, incomplete, centripetal enhancement, characterized by peripherally enhanced (extraocular muscles), and centrally unenhanced areas (optic nerve). Chorioretinal and anterior uveal regions showed a uniform, complete parenchymal enhancement. The contrast distribution of ROI 2 was rapid with progressive expansion from the area of the optic disk along the posterior curvature of the globe. In ROI 3 and ROI 4, a slightly biphasic pattern was observed with enhancement of the iris immediately before the ciliary body. The relative postcontrast intensity of ROI 1 was hypoenhanced compared to all other ROIs, whereas ROI 2, ROI 3, ROI 4 appeared qualitatively isoenhanced.

### 3.6 Time point assessment

**Figure 5:**
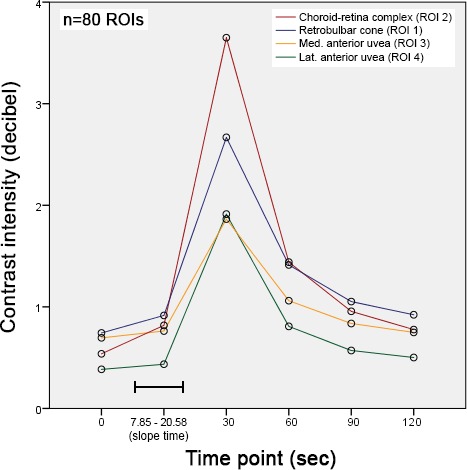
Temporal course of contrast intensity including data from 80 ROIs. Measurements at 30 and 60 sec were significantly higher compared with all other time points (*P* < 0.001). Slope time is expressed as range (sec) because intensity was analyzed from individual time points

Throughout the 120 sec evaluation period, contrast enhancement at the 30 and 60 sec time points differed significantly (*P* < 0.001) from all other time points when comparing measurements of all ROIs (Figure 5). Decibel differences between 0 sec and slope time (*P* = 0.179) and between slope time and 90 sec (*P* = 0.267), as well as 120 sec (*P* = 1.000), were not statistically significant. In 80% of all included ROIs (64/80), TTP was less than 25 sec, while in 11/80 (13.75%) PI was reached after 25 sec and in 5/80 (6.25%) TTP took 30 sec or longer (Table 3).

**Table 3:** Time to peak (TTP) distribution (n = 80 ROIs)

Time window (TTP)	Distribution of ROIs
<20 sec	18/80 (22.5%)
20.00‐24.99 sec	46/80 (57.5%)
25.00‐29.99 sec	11/80 (13.75%)
30.00‐34.99 sec	3/80 (3.75%)
35.00‐39.99 sec	2/80 (2.5%)

n, number of ROIs; ROI, region of interest.

## 4 DISCUSSION

Ultrasonography of the eye and orbit is routinely performed without benefit of contrast enhancement. This study confirmed the previously reported suitability of CEUS in canine eyes.^8^, ^9^, ^10^, ^20^ In various studies, SonoVue® has been administered intravenously at 0.03 mL/kg,^20^ 0.05 mL/kg,^10^ and 0.1 mL/kg^9^ bodyweight to examine uveal neoplasias, intraocular masses, and retinal detachment versus vitreous membrane in cats and dogs, respectively. In our setting, a dosage of 0.03 mL/kg bodyweight enabled visualization of intra‐ and extraocular perfusion in conscious beagles. Despite reported pathologic findings,^9^, ^10^, ^20^ physiologic characteristics of ocular CEUS are lacking.

The current study verified quantitative blood flow assessment simultaneously in four ROIs on the same horizontal plane.^15^ Time‐intensity curves revealed excellent or good quality in initial CEUS examinations in 8/10 (80%) animals. Even in painful patients, ocular ultrasonography is predominantly well‐tolerated using only topical anesthetics.^7^, ^8^ Based on the temporal perfusion characteristics in this study, the first 60 seconds after bolus injection are the most valuable diagnostic window. The fact that more than 90% of all ROIs reached their intensity peak in less than 30 seconds followed by a descending slope stresses the relevance of the first minute. In the authors’ experience, reduction of recording time by half increases the probability that most dogs remain cooperative, facilitating CEUS measurements.

The majority of patients undergoing ocular diagnostic imaging are affected unilaterally.^6^, ^7^, ^26^ This is the first study in veterinary ophthalmology evaluating whether order of bolus injections or laterality of the eyes influences contrast enhancement. Regarding both objectives, no statistically significant differences in any perfusion parameter or in any ROI were ascertained. These results indicate that CEUS provides repeatable, independent perfusion parameters of intra‐ and extraocular target tissues in healthy dogs and that the opposite eye can serve as a reliable in vivo reference. A standardized CEUS protocol can help to detect vascular alterations and pathologies in clinical cases.

A slightly longer TTP was observed in the second eye, although there was no statistical significance. It can be speculated that adaption of the dogs to the CEUS procedure contributed to a lower heart rate, but the correlation of hemodynamic changes and perfusion parameters was not scope of our study. Interestingly, in human renal carcinomas, TTP was shorter compared to normal kidneys^27^ and malignant breast cancer was differentiated from benign lesions by a significantly reduced TTP.^28^ Saracco et al^29^ recently showed that TTP can indicate responsiveness to neoadjuvant chemotherapy in invasive breast cancer. The diagnostic value of TTP for objective, quantitative diagnosis and monitoring in canine ocular and orbital neoplasias deserves further study. Measurements of AUC were consistently higher with the second bolus injection in all ROIs, except for AUC at ROI 3. A similar effect with repeated contrast injections has been found in the kidney and spleen in healthy cats.^30^ The causality of this phenomenon remains subject of discussion.

Time to peak of our pooled data was similar to preliminary results in the normal feline uvea (TTP: choroid: 15.16 sec, iris: 20.42 sec, ciliary body: 21.19 sec) evaluated by sulfur hexafluoride.^20^ It appears possible that PI is reached earlier in cats due to the shorter distance for microbubbles from the injection site to the heart and the eye. Hong et al^8^ used perfluorobutane under intramuscular zolazepam/tiletamine and medetomidine anesthesia. Time to initial upslope (TTU) and TTP in the posterior segment and retrobulbar region (14.21 ± 1.63; 23.38 ± 3.85 sec), the region of the ciliary body (20.67 ± 4.42; 27.18 ± 5.30 sec), and the region of the iris vessels (19.53 ± 3.22; 31.60 ± 2.97 sec) resulted in delayed contrast enhancement compared to slope time and TTP in the present study and lasted over 5 minutes in the posterior segment.^8^ Perfluorobutane dispersion has a longer elimination half‐life than sulfur hexafluoride. Additionally, TTU and TTP were influenced by cardiovascular response to anesthetic protocol.^31^, ^32^, ^33^ It could be appreciated that the elimination of perfluorobutane gas in the recent study^8^ was also altered by a reduced respiratory rate. In agreement with other reports,^8^, ^20^ our data confirm that slope time and TTP are relatively constant parameters which vary between posterior and anterior ocular structures in a predictable manner. The clinical significance of perfusion changes quantified by TTP merits future investigations in veterinary ophthalmology.

Color Doppler, power Doppler, and CEUS have been compared regarding their ability to detect vascular signals.^8^ These qualitative features, which might be altered by neoplastic angiogenesis, lack pivotal assessment of the entire parenchymal perfusion in each ROI. The qualitative assessment in this study revealed consistent enhancement patterns for the specific ROIs in all CEUS examinations. The ROI 2, ROI 3, and ROI 4 appeared isointense with subjective, qualitative evaluation, while quantitative assessment showed objective differences. Blood signals for the posterior segment and retrobulbar region were not significantly different when comparing Doppler techniques and CEUS.^8^ This underscores the importance of concurrent quantitative perfusion parameters. Enhancement patterns have been researched in human oncology patients to distinguish between malignant and benign lesions.^34^, ^35^ However, TIC parameters are the most reliable diagnostic tool.^27^, ^28^, ^34^

In canine eyes, time‐intensity curve parameters were obtained from 9 to13 mm circled areas at the above‐mentioned three ROIs with broadly defined localizations.^8^ The current study determined blood flow per tissue unit to objectively quantify physiologic perfusion parameters. The analyzed areas in CEUS of thoracic or abdominal organs commonly exhibit standardized region size and shape. On the one hand, this appears less suitable for delicate intraocular structures, on the other hand, the retrobulbar cone includes various tissues and blood vessels and applying ROIs exclusively to a target structure is challenging, when coping with head and ocular movements. Hence, perfusion parameters of specified ROIs containing the maximal representative tissue cross‐section were obtained and analyzed as absolute values and in relation to individual region sizes. The consensus between medial and lateral anterior uveal regions justifies accuracy of our measurement concept.

Peak intensity and AUC revealed the highest values per mm^2^ in the anterior uveal regions even though the choroid‐retina complex measurements were not significantly lower. These ROIs were isoenhanced on qualitative evaluation where the subtle differences between ROI 3, ROI 4, and ROI 2 were not apparent. Peak intensities in this study cannot be compared directly with previous results^8^ due to different definition, size, and shape of the ROIs. Furthermore, Hong et al^8^ did not determine an AUC. We believe that our assessment is more applicable and therefore beneficial for clinical purposes.

Noninvasive ocular CEUS using sulfur hexafluoride was well‐tolerated in all dogs based on clinical assessment only. Previous results determined CEUS as a safe examination technique.^36^ Additionally, no short‐term ophthalmic side effects were observed in the present study. Nevertheless, off‐label use may cause interactions between insonated microbubbles and cells.^37^ Adverse bioeffects to the chorioretinal microvasculature might have serious consequences and avoidance of high mechanical indices and long exposure times are advised. The expense of CEUS equipped ultrasound units and contrast medium can be compensated by planning several consecutive patients with different applications since most cases are elective procedures. Particularly in older patients with ocular or orbital neoplasia and if the owner's prefer to avoid sedation or anesthesia, CEUS is a promising diagnostic option.

While this study provides essential insight into intra‐ and extraocular perfusion assessment, some limitations must be addressed. Due to the relatively small sample size reference range of perfusion parameters should be interpreted with caution, especially when different equipment or contrast agents are used. All procedures were performed by one clinician to ensure high reproducibility and avoiding inter‐examiner variability. Clinical situations not necessarily guarantee this level of standardization, and operator dependent variation is expected. The data from our study cannot be extrapolated to the general canine population, but provide an indicative guideline. Further research with other breeds, ages, or female dogs is required to corroborate our results.

Consideration must be given to the fact that measurements without chemical restraint may be less precise than under sedation or anesthesia. An experienced sonographer keeping steady transducer contact to the eye and motion correction in QLAB Release 10.7® can resolve this problem. However, TICs should be assessed to exclude poor quality.

Conventional ultrasonography is frequently required to evaluate intraocular and retrobulbar pathologies, although its accuracy varies among diseases.^38^ B‐mode and Doppler techniques may be at risk of misdiagnoses^7^, ^38^ and artifacts.^39^ Computed tomography (CT) in dogs is often limited to differentiation of neoplastic vs nonneoplastic lesions.^5^ Ultrasonographic and CT findings of bony lysis are suggestive for orbital malignancy,^5^, ^7^ but chronic inflammation or compression due to benign conditions can cause similar features. The diagnostic property of noninvasive, quantitative CEUS to differentiate between malignant and benign tumors is vital for appropriate treatment^4^, ^7^, ^40^ and prognosis in canine uveal and orbital neoplasias. Moreover, CEUS enables detailed presurgical perfusion assessment in case of exenteration. Further investigations correlating CEUS parameters to histopathology are warranted in canine ophthalmology.

This study provides knowledge of the physiologic perfusion of ocular and orbital parenchyma to the level of capillaries. In conclusion, CEUS parameters of the opposite eye can serve as an in vivo reference for the contralateral target tissue. The first minute of the CEUS protocol shows the most significant blood flow information in conscious dogs.

## CONFLICTS OF INTERESTS

The authors have no conflicts of interests to declare.
